# Chemical vs. Enzymatic Refining to Produce Peanut Oil for Edible Use or to Obtain a Sustainable and Cost-Effective Protector for Stored Grains against *Sitophilus zeamais* (Coleoptera: Curculionidae)

**DOI:** 10.3390/foods11091224

**Published:** 2022-04-24

**Authors:** Monica Macaluso, Priscilla Farina, Linda Rossi, Alessandro Bianchi, Francesca Venturi, Rodrigo Daniel Chiriboga Ortega, Stefano Bedini, Barbara Conti, Luca Guidi, Angela Zinnai

**Affiliations:** 1 Department of Agriculture, Food and Environment, University of Pisa, 56124 Pisa, Italy; monica.macaluso@phd.unipi.it (M.M.); priscilla.farina@phd.unipi.it (P.F.); alessandro.bianchi@phd.unipi.it (A.B.); stefano.bedini@unipi.it (S.B.); barbara.conti@unipi.it (B.C.); angela.zinnai@unipi.it (A.Z.); 2 SALOV S.p.A., Via di Montramito, 1600, 55054 Massarosa, Italy; linda.rossi@salov.com; 3 Interdepartmental Research Center, Nutraceuticals and Food for Health, University of Pisa, 56124 Pisa, Italy; luca.guidi@salov.com; 4 Department of Biotechnology, Universidad Técnica del Norte, Ibarra 100105, Ecuador; rodrigolive@hotmail.es

**Keywords:** enzyme, refined process, phospholipids, wheat, maize weevil, foodstuff pest, toxicity

## Abstract

Among the various existing techniques, enzymatic degumming represents a process that is establishing itself as a valid alternative to the more classic chemical processes. Moreover, vegetable oils of various origins have been gaining more consideration as sustainable and affordable protectants for cereals and pulses against the attack of several insect pests. *Sitophilus zeamais* (Motschulsky) (Coleoptera: Curculionidae) is one of the key pests of cereal crops in the field and in stored and processed cereal products. Based on these highlighted issues, the overall aim of this research was twofold: (i) firstly, the effectiveness of the enzymatic degumming process was evaluated through the use of three different enzymes in order to verify the possible industrial application within the SALOV company as an alternative to the conventional chemical process; (ii) in a second phase, the possible use of the innovative refined oils was explored for sustainable stored grain protection towards *S. zeamais*. The results obtained confirm the strong possibility of applying the enzymatic process, which is innovative and, in a chemical way, more sustainable than the classical one. Regarding the toxicity towards *S. zeamais*, the crude peanut oil and the chemically refined peanut oil had lower LC_50_ values (1.836 and 1.372 g kg^−1^, respectively) than the oils rectified through enzymatic degumming (LC_50_ from 2.453 to 4.076 g kg^−1^), and, therefore, they can be suggested as sustainable stored grain protectants.

## 1. Introduction

The refining process is aimed at removing all those compounds, naturally contained in crude oil, which cause defects in the nutritional health, qualitative, and organoleptic–sensorial characteristics or during storage [1,2].

Within crude oil, it is possible to distinguish between two macro-categories of compounds, consisting of the fat-soluble fraction and the non-fat-soluble fraction [3,4]. The non-fat-soluble fraction includes compounds such as fibers, proteins, residual moisture, and impurities of various kinds. The liposoluble fraction, on the other hand, includes all those compounds with non-polar (or lipophilic) parts within their molecule, such as glycerides, free fatty acids, phospholipids, glycolipids, waxes, sterols, tocopherols, pigments, hydrocarbons (aldehydes and ketones), traces of metals, etc. [4,5].

As part of the refining of vegetable oils, the degumming phase (effective removal of phospholipids) is a fundamental operation for the success of the process, which can significantly affect the final yield and, consequently, the proceeds in economic terms. With the aim of increasing the yield of the process, as well as its sustainability, among the various existing techniques, enzymatic degumming represents a process that is establishing itself as a valid alternative to the more classic chemical processes [6,7,8]. The enzymatic process for degumming has fairly recent origins, as it was firstly developed in 1993 by Röhm GmbH, with the name of EnzyMax^®^ [9], patented by Aalrust et al. [10]. Subsequently, this type of process was resumed by Novozymes towards the end of the 1990s, through the development of an enzyme of microbial origin [11]. Since then, numerous types of enzymes have been developed, passing from the first- to the second-generation ones, up to the third-generation enzymes in recent years and, to the best of our knowledge, research in this topic is still far from complete.

In the field of vegetable oils, peanut oil, *Arachis hypogaea* L. (Fabaceae), is the one that has aroused the greatest interest since the extraction yield is quite high, around 40–50%, and this is one of the reasons that its production is particularly advantageous [12].

Moreover, vegetable oils of various origins have been gaining more consideration as sustainable and affordable protectants for cereals and pulses against the attack of several insect pests of stored foodstuffs [13,14,15,16,17,18,19]. Such insects already consume and damage up to 20% of the yielded grains, and the percentage is supposed to increase due to the rising temperatures [20,21]. Their management has been lately attempted using eco-friendly substances such as inert dusts [22,23,24] and essential oils [25,26,27,28] and employing predators and parasitoids [29,30]. Despite this, phosphine and synthetic insecticides are still widely applied [31,32], and these chemical solutions cause resistance development even in field populations, with severe drawbacks for the environment and non-target species [33,34].

*Sitophilus zeamais* (Motschulsky) (Coleoptera: Curculionidae), known as the maize weevil, is one of the key pests of cereal crops in the field and in stored and processed cereal products [35,36] throughout warm, humid regions around the world.

Based on these highlighted issues, the overall aim of this research was twofold: (i) firstly, the effectiveness of an innovative enzymatic degumming process was evaluated through the use of three different enzymes recently proposed at industrial scale for different purposes (“ROHALASE^®^ PL-XTRA” by AB Enzymes (Darmstadt, Germany), “Quara^®^ LowP” of Novozymes ((Monza, Italy)), and “Purifine^®^ PLA1” by DSM) in order to verify the possible industrial application within the SALOV company as an alternative to the conventional chemical process; (ii) in a second phase, the possible use of the innovative refined oils was explored in comparison with crude peanut oil and a traditionally refined one for sustainable stored grains’ protection towards *S. zeamais*.

## 2. Materials and Methods

### 2.1. Raw Material

The oil used for the experimentation was a crude peanut oil supplied by SALOV S.p.A. (Massarosa, Italy), obtained through solvent extraction. All the parameters (Table S1), necessary to define the enzymatic activities, were previously evaluated, such as, in particular, the free acidity and phosphorus/phospholipids present.

### 2.2. Refining Process

**Figure 1:**
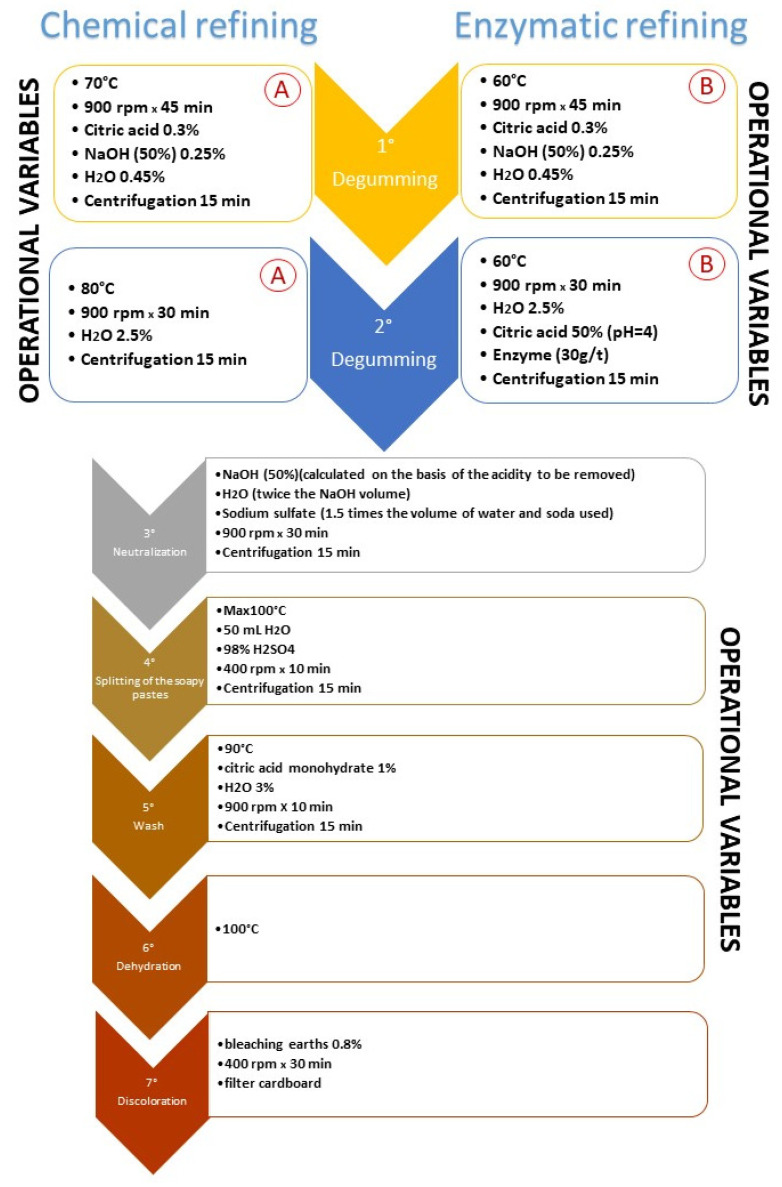
Refining process. Comparison between chemical refining (1° A and 2° A characterize the chemical process, usually used in SALOV) and enzymatic refining (1° B and 2° B characterize the enzymatic process). All the other phases (3°–7°) are common to both systems.

As reported in Figure 1, the experimental process (enzymatic) differed from the traditional one (chemical) mainly in the operating conditions adopted during degumming (phase 1 and 2 of the whole process), while the different experimental lines continued in the same way, starting from phase 3 until the end of the refining process.

### 2.3. Enzymes

The enzymes used for degumming were:

1. Quara^®^ LowP, produced by Novozymes (Monza, Italy) and distributed in Italy by Univar Solutions;
2. ROHALASE^®^ PL-XTRA, produced by AB Enzymes and distributed in Italy by Barentz;
3. Purifine^®^ DSM, produced by DSM Food Specialties B.V. (Delft, The Netherlands).

These are phospholipases belonging to different classes:

1. Quara^®^ LowP is a PLA1, having hydrolytic activity on fatty acids in sn-1 position and partial activity also tested on fatty acids in sn-2 position;
2. ROHALASE^®^ PL-XTRA is a PLA2, having exclusive hydrolytic activity on the fatty acids in the sn-2 position;
3. Purifine^®^ DSM is a PLA1, a (lyso-) phospholipase that cuts the sn-1 position of a (lyso-) phospholipid.

As regards the operative parameters applicable for the three enzymes, these are substantially comparable.

Based on the refining process carried out in the peanut oil processing plant, the operating parameters chosen for experimentation in the enzymatic degumming phase were:

- Enzyme dosage equal to 30 g/t of crude oil;
- Temperature of the enzymatic degumming phase equal to 60 °C;
- Water dosage in the enzymatic degumming phase equal to 2.5%;
- 50% citric acid dosage in the enzymatic degumming phase equal to 650 g/t oil (pH mixture with oil ≅ 4);
- Contact time of the enzymatic degumming phase equal to 30 min.

### 2.4. Samples

- RA—Conventional degumming SALOV;
- RD—Rectified with Purifine^®^ DSM;
- RQ—Rectified with Quara^®^ LowP;
- RR—Rectified with ROHALASE^®^ PL-XTRA;
- S—Crude peanut oil.

### 2.5. Reagents

The reagents used in the various phases of the experiment were:

- Citric acid (C_6_H_8_O_7_) at 50% by Bionova;
- 50% NaOH by Subolab GmbH;
- Drinking tap water;
- Ethanol blend—diethyl ether by Applichem 1:1 *v/v* + 15 mg/L phenophthalein by Biopharm;
- NaOH 0.1 N by Sigma-Aldrich;
- 10° Bé solution of sodium sulphate (Na_2_SO_4_) by Sigma-Aldrich;
- 98% sulfuric acid (H_2_SO_4_) by Sigma-Aldrich;
- Citric acid (C_6_H_8_O_7_) monohydrate by Bionova;
- Bleaching earths (CC160 Carbonitalia srl).

### 2.6. Free Fatty Acidity

Free fatty acidity was determined as previously described by Flori et al. [37].

To evaluate the acidity value necessary to verify the correct functionality of the enzyme, we used the indications reported by the manufacturers of the enzymes used in this test.

The total expected increase in free acidity must be between:

(1) $$\frac{0.1\times ppm\;P\;initials\;crude\;oil}{100}<total\;increase\;\%\;FFA<\frac{0.7\times 1.8\times ppm\;P\;initials\;crude\;oil}{1000}$$

However, only approximately 25–35% of this increase will end up in the degummed oil, while approximately 75–65% will end up in the aqueous phase together with the tires. In degummed oil, therefore, the expected real increase in free acidity must be between:

(2) $$0.25\times \frac{0.1\times ppm\;P\;initials\;crude\;oil}{100}<total\;increase\;\%\;FFA<\frac{0.7\times 1.8\times ppm\;P\;initials\;crude\;oil}{1000}\times 0.35$$

The tests were conducted in triplicate.

### 2.7. Color Measurement

The color was measured using a Lovibond^®^ tintometer (Model F, Greenwich, UK). The sample color was matched by adjusting the red (a*) and yellow (b*) values while keeping the blue unit fixed at 0.1. The corresponding color units were recorded. The hue angle and chroma values were determined using the formulae, tan − 1(b/a) and (a2 + b2)1/2, respectively, where a = red unit, b = yellow unit [38]. The tests were conducted in triplicate.

### 2.8. Phospholipids

The quantitative determination of the phospholipids was carried out using the SOBIODA Phospholipids colorimetric enzyme kit [39]. This method exploits the hydrolysis action of phospholipase D, and the choline released is subsequently oxidized by choline oxidase (CHO) to betaine with the simultaneous production of hydrogen peroxide. In the presence of peroxidase, hydrogen peroxide forms quinonemin with 4-Aminophenazones (4-AP) and dichlorophenol. The intensity of the color formed is proportional to the concentration in the phospholipids. The spectrophotometric measurement is recorded at 505 nm. The amount of phospholipids is determined according to the following equation:

((Absorbance(sample))/(Absorbance (Standard))) × 300(concentration of standard) = [mg/dL](3)

The tests were conducted in triplicate.

### 2.9. Sitophilus Zeamais Rearing Conditions

The *S. zeamais* rearing is permanently maintained at the Department of Agriculture, Food and Environment of the University of Pisa. The species is reared according to Romani et al. [28], with minor changes. The whole life cycle is completed in polyethylene boxes (27 cm × 20 cm × 11 cm) closed with a mesh lid for aeration, provided with grains (corn and soft wheat, renewed two times a month) for nutrition and reproduction. The cages are kept inside a closed climatic chamber (KW Srl., Siena, Italy), in the dark, at 65% R.H. and 26 °C. The adult weevils of the same age employed in the toxicity test are obtained by sifting the grain to move all the specimens away and waiting 24 h for the emergence of the new adults to be tested.

### 2.10. Toxicity of the Oils on Sitophilus Zeamais

The toxic effect of the SALOV crude peanut oil (S), rectified A (RA), rectified D (RD), rectified Q (RQ), and rectified R (RR) oils was tested on *S. zeamais*. The experiment was conducted in cylindrical glass chambers (volume 330 mL) filled with 100.0 g of soft wheat kernels, *Triticum aestivum* L. (Poaceae). Before being used, wheat was kept in a freezer at −20 °C for 48 h to eliminate any accidental infestation. After the return to room temperature, the wheat was poured with 0.0 (control), 50.0, 100.0, 150.0, 200.0, 300.0, and 400.0 µL of one of the five oils investigated (corresponding to the doses of 0.0 (control), 0.46, 0.92, 1.38, 1.84, 2.76, and 3.68 g kg^−1^) and thoroughly shaken for 5 min to evenly spread the treatments. Then, we put twenty unsexed adults (0–3 days old) in each chamber. The surviving insects were not subjected to further tests. In the end, the chambers were closed with tinplate screw lids (diameter 6.5 cm) and kept inside a closed climatic chamber (KW Srl., Siena, Italy), in the dark, at 65% R.H. and 26 °C. The test was replicated four times for each dose of each oil involved in the trial, including the control samples. The entire assay lasted 15 days. Mortality was checked four times (after 3, 6, 9, and 12 days from the beginning), gently sifting the treated wheat. When counting the dead weevils, we considered the death-feigning habit (thanatosis) of the species, observing the weevils for up to five minutes.

### 2.11. Statistical Data Processing

Statistically significant differences among oils’ physical–chemical parameters were determined by analysis of variance (ANOVA) using a significance level ρ = 0.05. The data were processed by a special software (CoStat, Cohort 6.0), using Tukey’s test and a completely randomized analysis.

Insects’ mortality values (%) were corrected using Abbott’s formula [40] to consider the natural mortality in the control samples. The relative toxicity of the oils was assessed, using probit analysis [41,42], by calculating the median lethal concentration (LC_50_). For each toxicity test, a probit model was built for the five oils. The fitness of the probit model [PROBIT(p) = Intercept + BX; where PROBIT(p) is the cumulative probability estimates, B is the slope of the model, and X is the oil dose transformed using the base 10 logarithm (covariate)] was tested through the Pearson goodness-of-fit test. Differences between LC_50_ values were assessed by relative median potency (rmp) estimates. Differences were considered significant if the rmp 95% confidence interval did not include 1. Statistical analyses were performed via SPSS 22.0 software (IBM SPSS Statistics, Armonk, New York, NY, USA).

## 3. Results and Discussion

Looking at Table 1, it is evident that the enzymatic degumming phase, with a different situation for the Purifine^®^ DSM, caused a slight increase in the free acidity of the oil (Table 1), an element that testifies to the correct functioning of the enzymes [6].

As a consequence of the enzymatic activity, the percentage of free fatty acids (acidity) present in the degummed oil increases compared to the starting acidity of the crude oil, as this indicates that the phospholipids have actually been hydrolyzed, causing the detachment of the fatty acids free from the glycerol skeleton [8].

Based on the analysis of crude peanut oil used for this experiment, the starting value of phospholipids was approximately 127.11 ppm in phosphorus (P). The expected free acidity increase in the degummed oil is therefore equal to:

(4) 0.032<expected increase %FFA<0.056

The parameters of temperature and contact time used during the enzymatic degumming phase, equal to 60 °C and 30 min, respectively, are for this reason suitable results, testifying to the possibility of applying this process while maintaining the operating parameters and the phases of processing currently used in industrial practice [6].

Furthermore, as regards the acidity of the neutral oil (Table 1) obtained after the neutralization phase, this is completely comparable in the case of the control tests and enzymatic degumming and respects the legal limits set by the current legislation for seed oils (DPR 22/12/1954 n. 1217 and subsequent amendments and additions, Law 27/01/1968 n. 35 and subsequent amendments and additions) [43].

Finally, the acidity of the oleins was also recorded (Table 1) following the splitting phase of the soapy pastes. An increase in the acidity of the oleins obtained in the case of enzymatic tests compared to the control was recorded. This amount is reasonable as a result of the increase in acidity in the enzymatically degummed oil and, therefore, of the greater amount of free fatty acids present within the soap pastes themselves.

Regarding the physical parameters, fundamental for the evaluation of process yields, we can note from Table 2 that the weight of degummed oil obtained from the first degumming phase was greater in the case of the enzymatic tests (RQ and RR) than in the control: this may be due to the use of NaOH in the RA test, which, albeit in a reduced dosage, causes the partial formation of soaps and therefore a loss of oil due to their emulsifying properties [9]. For the same reason, also considering the flocculating action allowed by the soda, the weight of the tires separated from the first phase was higher in the case of the control tests, as was reasonable to expect.

The weight of degummed oil obtained from the second degumming phase was higher in the case of the enzymatic tests compared to the control: this clearly shows the greater effectiveness of the enzymatic process compared to the water degumming applied in the second phase of the RA process. The greater amount of oil obtained is a direct consequence of the hydrolysis of the phospholipids present in the oil by the enzymes used, which allows for greater hydration and, therefore, a more effective separation from the oil [7].

The weight of the gums separated by the second degumming phase was lower in the case of the enzymatic tests compared to the control, as well as the relative volume: this is always explained as a consequence of the activity of the phospholipases, as the hydrolysis of the phospholipids causes the loss of their emulsifying abilities, and this causes them to retain a smaller amount of oil inside them [8]. As a consequence of this phenomenon, not only is the separation of a greater quantity of degummed oil possible in the process, but the volume of the eliminated rubbers and their viscosity are also significantly reduced, thus guaranteeing considerable operational advantages.

As for the results recorded following the neutralization phase (Table 2), the weight of the degreased soap pastes separated from the neutral oil was greater in the case of enzymatic tests than in the control. The figure is reasonable as a result of the increase in acidity in the enzymatically degummed oil, since the greater amount of free fatty acids present necessarily leads to the formation of a greater amount of soapy pastes. Furthermore, the measurement of the quantity of oil retained in the soap pastes (Table 2) shows a relative decrease in the weight of neutral oil retained in the soap pastes in the case of the enzymatic tests compared to the RA.

Upon completion of the neutralization phase, the weight of the oleins (Table 2) was also reported. Moreover, in this case, there was an increase in the weight of the oleins obtained in the case of the enzymatic tests compared to the RA. The figure is reasonable as a result of the increase in acidity in the enzymatically degummed oil and, therefore, of the greater amount of free fatty acids present within the soap pastes themselves.

Table 3 shows the values recorded following the colorimetric test (Lovibond) on the discolored oil samples obtained in the various tests. The values obtained are perfectly in line with company requirements, as they are even very close to those typically obtained in peanut oils at the end of the refining process (and therefore already deodorized) carried out in the company (SALOV).

It is possible to note how the values inherent in the parameter of yellow (Y) are substantially comparable, while a clear decrease in red (R) has been recorded, which is the most problematic parameter in the case of peanut oil refining, as it is much more difficult to break down. These data allow us to confirm the influence of the enzymatic degumming phase also on the subsequent phases of the refining process. This can be justified in reference to the higher phosphorus (P) abatement recorded in the enzymatic processing (Table 4), which allowed us to obtain a residual rubber content in the neutral oil lower than the control, ensuring greater efficiency of the bleaching earths themselves, as the residual gums are adsorbed on the surface of the earths together with the pigments to be eliminated, reducing their efficiency.

As shown in Table 5, the theoretical yield of the degumming phase underwent an increase of approximately 0.60% in the case of Quara^®^ LowP and approximately 0.57% in the case of ROHALASE^®^ PL-XTRA. This is a significant increase that fully justifies the possibility of applying the enzymatic process in industrial practice. The higher costs of the enzymatic process compared to RA, attributable in the first place to the cost of the enzyme and secondly to the slight increase in the dosages of citric acid and NaOH required, respectively, for the second degumming phase and the subsequent neutralization phase, are in fact caused by being fully compensated by the increase in yield obtained. Furthermore, the rubbers separated by the second degumming phase are lower in terms of volume and significantly more fluid than those separated in superdegumming: this entails greater ease in their disposal, and, at the same time, less maintenance of the machines used and, therefore, less downtime necessary to clean up the plant, which actually increases daily revenues.

As regards the subsequent refining phases analyzed, the theoretical yield of the neutralization phase showed a slight increase, compared to the control, in the case of processing by the enzymatic route, despite the separation of a much greater quantity of soapy pastes. These data can be justified as a further consequence of the enzymatic activity during the degumming phase: the reduction of phosphorus (Table 4) obtained in the degummed oil by the phospholipases, in fact, causes the gums to be eliminated almost completely with the degumming phase, thus avoiding their dragging into the subsequent neutralization phase. Consequently, in the case of enzymatic processing, a further increase in the emulsifying properties of the soapy pastes is prevented, as they also contain the residual gums present in the degummed oil, which would inevitably lead to a greater loss of neutral oil.

Similarly, a slight increase in the theoretical yield of the bleaching phase (Table 5) was recorded, compared to the control, in the case of enzymatic processing. Moreover, in this case, the data can be justified as a further consequence of the enzymatic activity during the degumming phase: the reduction of phosphorus (Table 4) obtained in the degummed oil by the phospholipases, in fact, causes the gums to be almost completely eliminated with the degumming phase [6], thus avoiding their dragging not only in the subsequent neutralization phase, but also in the discoloration phase. Consequently, in the case of enzymatic processing, the neutral oil obtained has a lower content of residual gums, which, in addition to hindering the efficiency of the bleaching earth more, would inevitably also lead to a greater loss of oil.

As shown in Table 4, the reduction in the recorded phosphorus (P) levels, a direct indicator of the phospholipid content in the oil, was higher in the case of enzymatic tests than in the control, as was expected.

The toxicity tests showed that all four rectified and the SALOV crude peanut oils are toxic towards the adults of *S. zeamais*. The most promising oils to be used as grain protectants are the crude peanut SALOV oil (S) and the oil rectified through conventional chemical degumming (RA). The three oils rectified through enzymatic degumming showed, instead, less toxicity towards *S. zeamais*. Indeed, the LC_50_ values ranged from 1.372 to 4.076 g kg^−1^ for the RA and RQ oils, respectively (Table 6). The rmp analysis indicated that the RA toxicity is significantly higher than that of the RD, RQ, and RR oils, while it is not significantly different from that of the S crude peanut oil (Table 6).

The control of *S. zeamais* using edible oils has been previously attempted by several authors at laboratory level. Law-Ogbomo and Enobakhare [17] obtained up to 93% of *S. zeamais* mortality 21 days after treating maize with 10 mL kg^−1^ of rubber seed, palm, and palm kernels oils. A much higher protection effect was obtained with cotton and *Brassica carinata* oils with, respectively, 100 and 90% of *S. zeamais* mortality after 20 days at 2.0 mL kg^−1^ maize [18]. Wale and Assegie [19] reported an LD_50_ of 2.04 mL of castor bean oil used on corn kernels against *S. zeamais*.

Peanut oil was successfully used as a protectant of numerous grains and pulses against several pests, including our target species and the closely related *Sitophilus granarius* (L.) and *S. oryzae* (L.). Ivbijaro [14] obtained 100% mortality of *S. zeamais* adults within 24 h using 20 mL kg^−1^ maize of peanut oil (a much higher concentration compared to that used in the present work) and within two and three days with, respectively, 5 and 10 mL kg^−1^ maize. On *S. oryzae*, 1 mL kg^−1^ maize of peanut oil caused the death of 33.3% of the tested weevils after one day and 95.8% after one week: higher doses (5 and 10 mL kg^−1^) led to complete mortality [15]. Obeng-Ofori and Amiteye [44] reported 93% and 100% mortality of *S. zeamais* with, respectively, 5 and 10 mL kg^−1^ maize of peanut oil after 24 h and similar toxicity with the oil blended with the synthetic insecticide pirimiphos-methyl. On *S. granarius*, peanut oil showed 65% mortality when used alone at 10 mL kg^−1^ wheat and complete mortality when mixed with pirimiphos-methyl after around two weeks [45]. To the best of our knowledge, this is the first attempt to use refined peanut oils as grain protectants against foodstuff insect pests.

Regarding the mode of action of vegetable oils, different hypotheses have been formulated during the past 40 years. As supposed by Law-Ogbomo and Enobakhare [17], mortality could be related to starvation, as the oil forms a film on the seeds that might prevent the insect from feeding. According to other authors, the insecticidal activity by contact could be due to the presence of toxic fatty acids (linoleic, oleic, palmitic, and stearic) in the oil composition [13,16,18]. Furthermore, the scanning electron microscope (SEM) examinations carried out by Aider et al. [46] revealed that the oil coating obstructs the insect’s spiracles, possibly causing suffocation.

## 4. Conclusions

Based on the experimental data collected, it was possible to confirm the validity of the innovative enzymatic degumming process proposed for peanut oil compared to the traditional one. Among the tested enzymes, based on the operating parameters used (temperatures and retention times), the experimentation carried out would seem to indicate the greater efficiency of the ROHALASE^®^ PL-XTRA enzyme given the lower levels of phosphorus recorded in the degummed oil, and its use in the corporate refining process (SALOV) is suggested. The possible application is justified both in reference to the higher oil yields obtained, the operational advantages deriving from the greater reduction of tires in the oil during degumming, and the economic feasibility of the process.

On the other hand, moreover, crude peanut oil and chemically refined peanut oil represent the best solutions for the protection of the grain and to fight *S. zeamais* in a sustainable and economic way. Further chemical and microscopy investigations could shed light on the mechanism(s) of action that causes the death of *S. zeamais* when using vegetable oils.

## Figures and Tables

**Table 1:** Acidity values measured in the experimental samples during the different phases of the pilot process (RA = conventional degumming SALOV, RQ = Rectified with Quara^®^ LowP, RR = Rectified with ROHALASE^®^ PL-XTRA, RD = Rectified with Purifine^®^ DSM).

Acidity Free (% Oleic Acid)	RA	RQ	RR	RD	*F*-Value	*R* ^2^
Starting acidity sample crude oil (%)	1.63 a ± 0.05	1.63 a ± 0.06	1.63 a ± 0.06	1.36 b ± 0.05	19.07	0.87
Oil acidity degummed (%)	1.47 b ± 0.09	1.73 a ± 0.06	1.71 a ± 0.03	1.12 c ± 0.03	196.62	0.99
Neutral oil acidity (%)	0.34 b ± 0.05	0.23 c ± 0.13	0.27 c ± 0.19	0.47 a ± 0.14	5.79	0.69
Olein acidity (%)	65.44 c ± 0.83	70.54 bc ± 2.83	76.15 ab ± 7.35	96.82 a ± 10.35	16.25	0.86

Data are expressed as mean ± SD; in the same line, the letters (a, b, c) indicate significant differences (*p* < 0.05) after the analysis of variance (ANOVA).

**Table 2:** Physical parameters measured in the experimental tests during the different stages of the process (RA = conventional degumming SALOV, RQ = Rectified with Quara^®^ LowP, RR = Rectified with ROHALASE^®^ PL-XTRA, RD = Rectified with Purifine^®^ DSM).

Physical Parameters	RA	RQ	RR	RD	*F*-Value	*R* ^2^
Sample starting weight crude (g)	600.06 a ± 0.06	600.08 a ± 0.11	600.05 a ± 0.04	600.00 a ± 0.02	0.63	0.19
Total weight of degummed oil obtained from the first phase of degumming (g)	587.68 b ± 1.04	589.24 a ± 1.13	589.22 a ± 1.17	585.94 c ± 1.12	1.80	0.40
Total weight of tires separated from the first degumming phase (g)	8.27 b ± 1.32	7.08 c ± 1.86	7.67 c ± 1.23	12.37 a ± 1.34	49.96	0.95
Total weight of degummed oil obtained from the second degumming phase (g)	575.87 b ± 2.01	579.52 a ± 2.35	579.29 a ± 5.62	580.71 a ± 5.62	1.04	0.28
Total weight of separate tires from the second phase of degumming (g)	10.48 a ± 1.21	8.94 b ± 1.34	9.94 b ± 0.75	8.18 c ± 0.84	3.59	0.58
Total volume of separate tires from the second phase of degumming (mL)	10.63 a ± 1.43	8.00 b ± 1.50	8.05 b ± 1.61	5.02 c ± 1.54	3.60	0.57
Total oil weight retained by the soap pastes (g)	1.04 b ± 0.63	0.59 c ± 0.32	0.70 c ± 0.20	2.06 a ± 0.30	25.74	0.90
Total weight of soap pastes degreased separated by the neutralization phase (g)	18.04 b ± 0.44	21.73 a ± 0.68	21.21 a ± 1.13	12.85 c ± 1.22	28.21	0.91
Olein weight obtained for 12 g of split soapy pastes (g)	3.98 b ± 0.17	4.31 a ± 1.11	4.35 a ± 1.04	4.30 a ± 1.03	1.63	0.37

Data are expressed as mean ± SD; in the same line, the letters (a, b, c) indicate significant differences (*p* < 0.05) after the analysis of variance (ANOVA).

**Table 3:** Values recorded with Lovibond test (colorimetric) carried out on the samples of discolored oil (RA = conventional degumming SALOV, RQ = Rectified with Quara^®^ LowP, RR = Rectified with ROHALASE^®^ PL-XTRA, RD = Rectified with Purifine^®^ DSM).

Colorimetric Test (Lovibond)	RA	RQ	RR	RD	*F*-Value	*R* ^2^
R	0.60 a ± 0.05	0.50 b ± 0.02	0.50 b ± 0.03	0.50 b ± 0.02	0.60	0.19
Y	4.20 a ± 0.30	4.17 a ± 0.07	4.07 a ± 0.35	4.12 a ± 0.30	0.16	0.06

Data are expressed as mean ± SD; in the same line, the letters (a, b) indicate significant differences (*p* < 0.05) after the analysis of variance (ANOVA).

**Table 4:** Comparison of phospholipid and phosphorus values with its reduction percentage compared to SALOV crude peanut oil (S = crude peanut oil, RA = conventional degumming SALOV, RQ = Rectified with Quara^®^ LowP, RR = Rectified with ROHALASE^®^ PL-XTRA, RD = Rectified with Purifine^®^ DSM).

Phosphorus and Phospholipids	S	RA	RQ	RR	RD	*F*-Value	*R* ^2^
Content in P (ppm)	127.11 a ± 5.22	69.12 b ± 0.13	60.22 d ± 0.11	52.17 e ± 0.14	63.52 c ± 0.12	497.02	0.99
Phospholipid content (ppm)	3289.17 a ± 16.22	1718.17 b ± 15.32	1503.66 c ± 11.13	1266.82 d ± 10.12	1613.17 b ± 12.32	11,135.08	0.99
Phosphorus abatement compared to crude oil (%)	-	45.62	52.62	58.95	50.02	-	-

Data are expressed as mean ± SD; in the same line, the letters (a, b, c, d) indicate significant differences (*p* < 0.05). - non detected. between the theses after the analysis of variance (ANOVA).

**Table 5:** Theoretical yields recorded in the various refining phases conducted on a pilot scale (RA = conventional degumming SALOV, RQ = Rectified with Quara^®^ LowP, RR = Rectified with ROHALASE^®^ PL-XTRA, RD = Rectified with Purifine^®^ DSM).

Theoretical Returns of Process Phases	RA	RQ	RR	RD
Yield of degumming phase (%)	95.97	96.57	96.54	96.74
Yield phase neutralization (%)	96.51	96.57	96.62	94.95
Washing phase yield (%)	98.50	98.64	98.79	98.26

**Table 6:** Median lethal concentration (LC_50_) of the rectified and the SALOV peanut oils to adults of the stored grain insect pest *Sitophilus zeamais*.

Oil	LC_50_ (95% FL)	Intercept ± SE	*p*-Value
RA	1.372 (1.025–1.858) a	−0.117 ± 0.055	0.03
RD	2.453 (1.796–3.429) b	−0.330 ± 0.060	<0.001
RQ	4.076 (2.930–5.952) c	−0.518 ± 0.060	<0.001
RR	3.153 (2.295–4.491) bc	−0.423 ± 0.060	<0.001
S	1.836 (1.367–2.525) ab	−0.224 ± 0.055	<0.001

LC_50_, median lethal concentration; FL, fiducial limits; Intercept, intercept of the probit regression equation. Model slope = 0.848 ± 0.068; Pearson Goodness-of-Fit Test, *χ*^2^ = 29.328, df = 26, *p* = 0.296. The letters (a, b, c) indicate significant differences according to rmp estimates for paired comparisons of the LC_50_ values. Data are given as g·kg^−1.^

## Data Availability

The data presented in this study are available on request from the corresponding author.

## Supplementary Materials

The following supporting information can be downloaded at: www.mdpi.com/10.3390/foods11091224/s1, Table S1: Major fatty acids in crude peanut oil (% wt).

## References

[1] Gargouri B., Zribi A., Bouaziz M. (2015). Effect of containers on the quality of Chemlali olive oil during storage. J. Food Sci. Technol..

[2] Babich I.V., Moulijn J.A. (2003). Science and technology of novel processes for deep desulfurization of oil refinery streams: A review. Fuel.

[3] Eftekhardadkhah M., Øye G. (2013). Correlations between Crude Oil Composition and Produced Water Quality: A Multivariate Analysis Approach. Ind. Eng. Chem. Res..

[4] Lercker G., Rodriguez-Estrada M.T. (2000). Chromatographic analysis of unsaponifiable compounds of olive oils and fat-containing foods. J. Chromatogr. A.

[5] Erol A.S., Özcan M.M., Er F. (2011). Composition and characteristics of some seed oils. Asian J. Chem..

[6] Dijkstra A.J. (2010). Enzymatic degumming. Eur. J. Lipid Sci. Technol..

[7] Yang B., Wang Y.H., Yang J.G. (2006). Optimization of enzymatic degumming process for rapeseed oil. J. Am. Oil Chem. Soc..

[8] Sampaio K.A., Zyaykina N., Wozniak B., Tsukamoto J., De Greyt W., Stevens C.V. (2015). Enzymatic degumming: Degumming efficiency versus yield increase. Eur. J. Lipid Sci. Technol..

[9] Mei L., Wang L., Li Q., Yu J., Xu X. (2013). Comparison of acid degumming and enzymatic degumming process for *Silybum marianum* seed oil. J. Sci. Food Agric..

[10] Cerminati S., Paoletti L., Aguirre A., Peirú S., Menzella H.G., Castelli M.E. (2019). Industrial uses of phospholipases: Current state and future applications. Appl. Microbiol. Biotechnol..

[11] Meinert H., Yi D., Zirpel B., Schuiten E., Geißler T., Gross E., Brückner S.I., Hartmann B., Röttger C., Ley J.P. (2021). Discovery of Novel Bacterial Chalcone Isomerases by a Sequence-Structure-Function-Evolution Strategy for Enzymatic Synthesis of (S)-Flavanones. Angew. Chem..

[12] Madhaven B.N. (2001). Final report on the safety assessment of peanut (*Arachis hypogaea*) oil, hydrogenated peanut oil, peanut acid, peanut glycerides, and peanut (*Arachis hypogaea*) flour. Int. J. Toxicol..

[13] Hill J., Schoonhoven A.V. (1981). Effectiveness of vegetable oil fractions in controlling the Mexican bean weevil on stored beans. J. Econ. Entomol..

[14] Ivbijaro M.F. (1984). Groundnut oil as a protectant of maize from damage by the maize weevil *Sitophilus zeamais* Motsch. Protect. Ecol..

[15] Ivbijaro M.F., Ligan C., Youdeowei A. (1985). Control of rice weevils, *Sitophilus oryzae* (L.), in stored maize with vegetable oils. Agric. Ecosyst. Environ..

[16] Kellouche A., Soltani N., Kreiter S., Auger J., Arnold I., Kreiter P. (2004). Biological activity of four vegetable oils on *Callosobruchus maculatus* (Fabricius) (Coleoptera Bruchidae). Redia.

[17] Law-Ogbomo K., Enobakhare D.A. (2006). Efficacy of rubber seed oil, palm oil and palm kernel oil as grain protectants against *Sitophilus zeamais* (Mots.) (Coleoptera: Curculionidae) in three maize varieties. J. Entomol..

[18] Gemechu F., Santiago D.R., Sori W. (2013). Laboratory evaluation of cotton (*Gossypium hirsutum*) and Ethiopian mustard (*Brassica carinata*) seed oils as grain protectants against maize weevil, *Sitophilus zeamais* M. (Coleoptera: Curculionidae). Afr. J. Agric. Res..

[19] Wale M., Assegie H. (2015). Efficacy of castor bean oil (*Ricinus communis* L.) against maize weevils (*Sitophilus zeamais* Mots.) in northwestern Ethiopia. J. Stored Prod. Res..

[20] Deutsch C.A., Tewksbury J.J., Tigchelaar M., Battisti D.S., Merrill S.C., Huey R.B., Naylor R.L. (2018). Increase in crop losses to insect pests in a warming climate. Science.

[21] Singano C.D., Mvumi B.M., Stathers T.E., Machekano H., Nyamukondiwa C. (2020). What does global warming mean for stored-grain protection? Options for *Prostephanus truncatus* (Horn) control at increased temperatures. J. Stored Prod. Res..

[22] Pierattini E.C., Bedini S., Venturi F., Ascrizzi R., Flamini G., Bocchino R., Girardi J., Giannotti P., Ferroni G., Conti B. (2019). Sensory quality of essential oils and their synergistic effect with diatomaceous earth, for the control of stored grain insects. Insects.

[23] Mortazavi H., Toprak U., Emekci M., Bagci F., Ferizli A.G. (2020). Persistence of diatomaceous earth, SilicoSec^®^ against three stored grain beetles. J. Stored Prod. Res..

[24] Salim M., Gökçe A., Naqqash N.M., Ersoy O. (2020). Insecticidal potential of native diatomaceous earth against *Sitophilus granarius* (Coleoptera: Curculionidae). Sarhad J. Agric..

[25] Bertoli A., Conti B., Mazzoni V., Meini L., Pistelli L. (2012). Volatile chemical composition and bioactivity of six essential oils against the stored food insect *Sitophilus zeamais* Motsch. (Coleoptera Dryophthoridae). Nat. Prod. Res..

[26] Bougherra H.H., Bedini S., Flamini G., Cosci F., Belhamel K., Conti B. (2015). *Pistacia lentiscus* essential oil has repellent effect against three major insect pests of pasta. Ind. Crops Prod..

[27] Bedini S., Bougherra H.H., Flamini G., Cosci F., Belhamel K., Ascrizzi R., Conti B. (2016). Repellency of anethole- and estragole-type fennel essential oils against stored grain pests: The different twins. Bull. Insectol..

[28] Romani R., Bedini S., Salerno G., Ascrizzi R., Flamini G., Echeverria M.C., Farina P., Conti B. (2019). Andean flora as a source of new repellents against insect pests: Behavioral, morphological and electrophysiological studies on *Sitophilus zeamais* (Coleoptera: Curculionidae). Insects.

[29] Adarkwah C., Obeng-Ofori D., Opuni-Frimpong E., Ulrichs C., Schöller M. (2019). Predator-parasitoid-host interaction: Biological control of *Rhyzopertha dominica* and *Sitophilus oryzae* by a combination of *Xylocoris flavipes* and *Theocolax elegans* in stored cereals. Entomol. Exp. Appl..

[30] Kamboh K.M.S., Aqueel M.A., Raza M.A. (2021). Evaluation of parasitic potential of *Anisopteromalus calandrae* (Howard) against *Callosobruchus maculatus* (F.), *Rhyzopertha dominica* (F.) and *Sitophilus oryzae* (L.) in grains treated with diatomaceous earths. Pak. J. Agric. Sci..

[31] Tsaganou F.K., Vassilakos T.N., Athanassiou C.G. (2021). Insecticidal effect of thiamethoxam against seven stored-product beetle species. J. Stored Prod. Res..

[32] Afful E., Elliott B., Nayak M.K., Phillips T.W. (2018). Phosphine resistance in North American field populations of the lesser grain borer, *Rhyzopertha dominica* (Coleoptera: Bostrichidae). J. Econ. Entomol..

[33] Lampiri E., Agrafioti P., Athanassiou C.G. (2021). Delayed mortality, resistance and the sweet spot, as the good, the bad and the ugly in phosphine use. Sci. Rep..

[34] Wakil W., Kavallieratos N.G., Usman M., Gulzar S., El-Shafie H.A.F. (2021). Detection of phosphine resistance in field populations of four key stored-grain insect pests in Pakistan. Insects.

[35] Suleiman R., Rosentrater K.A., Bern C.J. (2015). Evaluation of maize weevils *Sitophilus zeamais* Motschulsky infestation on seven varieties of maize. J. Stored Prod. Res..

[36] Ojo J.A., Omoloye A.A. (2016). Development and life history of *Sitophilus zeamais* (Coleoptera: Curculionidae) on cereal crops. Adv. Agric..

[37] Flori L., Macaluso M., Taglieri I., Sanmartin C., Sgherri C., Leo M.D., Ciccone V., Donnini S., Venturi F., Pistelli L. (2020). Development of Fortified Citrus Olive Oils: From Their Production to Their Nutraceutical Properties on the Cardiovascular System. Nutrients.

[38] Szabó R.T., Mézes M., Szalai T., Zajácz E., Weber M. (2016). Colour identification of honey and methodical development of its instrumental measuring. Columella J. Agric. Environ. Sci..

[39] Bacle A., Kadri L., Khoury S., Ferru-Clément R., Faivre J.-F., Cognard C., Bescond J., Krzesiak A., Contzler H., Delpech N. (2020). A comprehensive study of phospholipid fatty acid rearrangements in the metabolic syndrome: Correlations to organ dysfunction. Dis. Models Mech..

[40] Abbott W.J. (1925). A method of computing effectiveness of an insecticide. J. Econ. Entomol..

[41] Finney D.J. (1971). Probit Analysis.

[42] Bedini S., Muniz E.R., Tani C., Conti B., Ruiu L. (2020). Insecticidal potential of *Brevibacillus laterosporus* against dipteran pest species in a wide ecological range. J. Invertebr. Pathol..

[43] Legge 13 febbraio 1957, n. 12. Conversione in legge del decreto-legge 20 dicembre 1956, n. 1380, pubblicato nella Gazzetta Ufficiale n. 321 del 21 dicembre 1956, che proroga le disposizioni di cui al decreto—legge 2 febbraio 1956, n. 28, convertito con modificazioni, nella legge 27 marzo 1956, n. 162, ed apporta modificazioni all’art. 30 del testo unico delle disposizioni concernenti la disciplina fiscale della lavorazione dei semi oleosi e degli oli da essi ottenuti, approvato con decreto del Presidente della Repubblica 22 dicembre 1954, n. 1217. https://www.normattiva.it/uri-res/N2Ls?urn:nir:stato:legge:1957-02-13;12@originale.

[44] Obeng-Ofori D., Amiteye S. (2005). Efficacy of mixing vegetable oils with pirimiphos-methyl against the maize weevil, *Sitophilus zeamais* Motschulsky in stored maize. J. Stored Prod. Res..

[45] Tembo E., Murfitt R.F.A. (1995). Effect of combining vegetable oil with pirimiphos-methyl for protection of stored wheat against *Sitophilus granarius* (L.). J. Stored Prod. Res..

[46] Aider F.A., Kellouche A., Fellag H., Debras J.F. (2016). Evaluation of the bio-insecticidal effects of the main fatty acids of olive oil on *Callosobruchus maculatus* F. (Coleoptera-Bruchidae) in cowpea (*Vigna unguiculata* (L.)). J. Plant Dis. Prot..

