# Supplementary material for: Chemical vs. Enzymatic Refining to Produce Peanut Oil for Edible Use or to Obtain a Sustainable and Cost-Effective Protector for Stored Grains against Sitophilus zeamais (Coleoptera: Curculionidae)
Source: Foods. 2022 Apr 24;11(9):1224. doi: 10.3390/foods11091224 (PMC9104994; doi:10.3390/foods11091224)
Supplement: Supplementary file 1 [file foods-11-01224-s001.zip › foods-1658659-supplementary.pdf]

**Supplementary File:**

**Table S1.** Major fatty acids in crude peanut oil (% wt).

| Fatty Acid | Percentage |
| --- | --- |
| Palmitic | 12.4 ± 0.3 |
| Stearic | 4.7 ± 0.9 |
| Oleic | 52.3 ± 1.3 |
| Linoleic | 23.9 ± 0.9 |
| Arachidic | 1.2 ± 0.5 |
| Behenic | 2.9 ± 0.7 |
| Lignoceric | 1.9 ± 0.8 |
